# An Anomalous Configuration of Coronary Artery: A Cadaveric Study

**DOI:** 10.1155/2013/397063

**Published:** 2013-05-16

**Authors:** Rajani Singh

**Affiliations:** Department of Anatomy, AIIMS, Virbhadra Marg, Pashulok, Rishikesh 249201, India

## Abstract

Anatomical variations in relation to coronary artery and its branches will help cardiac surgeons for refining imaging techniques and coronary artery bypass grafting. A heart was detected with multiple anomalies of coronary arteries in a cadaver. The anomalies of coronary arteries in terms of origin, number of ostia, courses, and presence of myocardial bridges were described, and related clinical implications were highlighted in the present study. The knowledge of variant anatomy may be of paramount importance to anatomists for variant anatomy and to cardiac surgeon for proper diagnosis and treatment of cardiac ailments including radiologists to refine image interpretation.

## 1. Introduction

 Rate of coronary-artery-related diseases is increasing by leaps and bounds in modern times. The anatomy of coronary artery has recently been reemphasized in association with the use of coronary arteriography. The advances made in coronary arterial bypass surgeries and modern methods of myocardial revascularization make sound and complete knowledge of the normal and variant anatomy of coronary artery [1] indispensable and imperative. Thus the variant cardiac anatomy is of paramount importance for proper understanding and management of cardiac diseases.

The heart is supplied by two coronary arteries, that is, right coronary artery (RCA) and left coronary artery (LCA). RCA originates from anterior aortic sinus at the root of the ascending aorta and LCA from left posterior aortic sinus at the root of the ascending aorta. RCA after arising courses between pulmonary trunk and right auricle then travels in right coronary sulcus, then winds round the inferior border of heart, then runs over the inferior surface, and ends by anastomosing with circumflex branch of LCA. LCA after coursing between pulmonary trunk and left auricle divides into anterior interventricular artery and circumflex artery. 

In the present case RCA and LCA have aberrant courses along with separate ostium for ACA and anomalously located ostium for RCA. The trifurcated LCA after its origin is also covered by myocardial bridge. The clinical significance of this new configuration of coronary arteries with new variant of myocardial bridge makes this study of paramount importance in management of heart diseases for cardiac surgeons and variant anatomy for anatomists. Therefore the study has been carried out.

## 2. Case Presentation

During routine dissection of cadaver of 50-year-old female, the heart was detected to have unique combination of variant configurations of RCA, ACA, and LCA in relation to origin, course, and presence of myocardial bridge as elaborated below.

**Figure 1:**
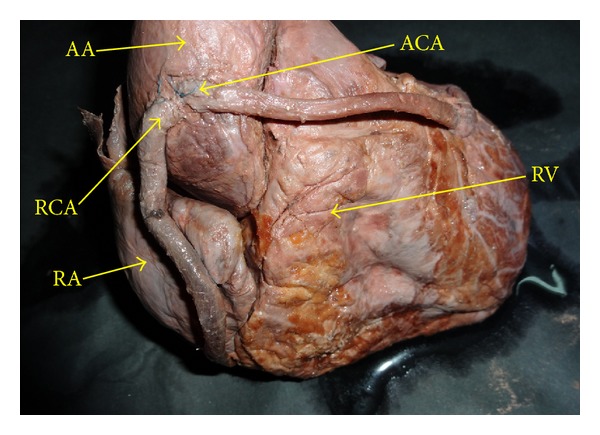
Showing high origin of coronary artery. AO: abdominal aorta, RCA: right coronary artery, ACA: accessory coronary artery, RA: right atrium, RV: right ventricle.

Firstly, RCA had *high origin* (anomalously located ostium) in the anterior part of ascending aorta measuring 3 cm above its root (Figure 1). 

This artery had an *aberrant course*. It descended down crossing aorta and right atrium and then entered the right anterior coronary sulcus for a very short distance. Now after crossing the inferior border it reached inferior surface travelling for 4 cm then sinking into myocardium.

**Figure 2:**
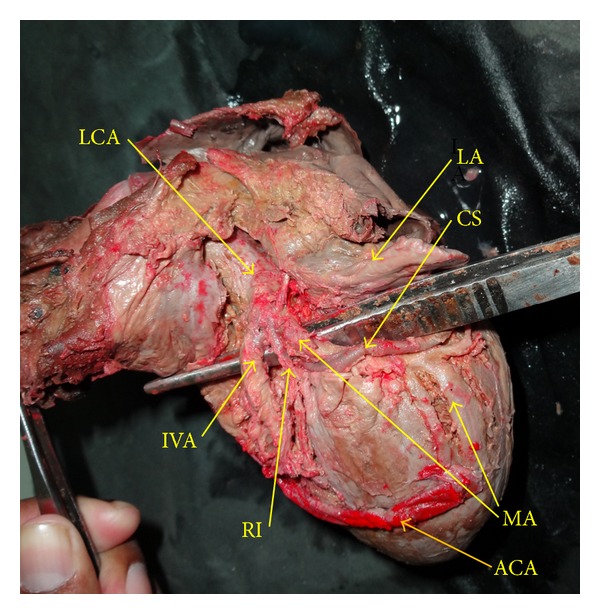
Showing trifurcation of left coronary artery. LA: left auricle, IVA: anterior interventricular artery, CS: coronary sulcus, RI: ramus intermedius, MA: marginal artery, ACA: accessory coronary artery.

Secondly, a *new variant *of* accessory coronary artery* (ACA) originated from the same level as RCA but from *separate ostium*. These ostia were separated by a distance of 0.2 cm. The path of ACA was oblique crossing firstly aorta, secondly infundibulum, thirdly left ventricle, and ending along the left margin of left ventricle (Figure 2). 

It formed arterial arcade with the diagonal artery and then with marginal artery. 

Thirdly, LCA originated from left aortic sinus and then divided into three branches (Figure 2): (1) Anterior interventricular artery, (2) ramus intermedius, and (3) marginal artery. All these three branches were covered by myocardial bridge and these branches were exposed by removing overlying myocardial fibres as displayed in Figure 2. The description of three branches of LCA, is appended below. 

The *anterior interventricular artery* ran underneath left auricle and then routed between the left auricle and root of pulmonary trunk. In its further course, it was embedded in fat which was removed by blunt dissection, and the artery was exposed (Figure 2). After emerging on the surface from myocardial bridge, it gave a branch as diagonal artery which was connected with ACA. The main anterior interventricular branch routed through anterior interventricular groove ending near the inferior border. 

*Ramus intermedius artery* appeared after passing out from myocardial bridge on the surface between the anterior interventricular artery and marginal artery. Advancing further by 2 cm on the surface of left ventricle and then entered into the anterior wall of left ventricle.

*Marginal artery* after passing through myocardial bridge appeared on the surface between the left auricle and left ventricle. Instead of entering into left anterior coronary sulcus, it took a turn, travelling along the left margin and ended by forming an arterial arcade with ACA. It gave two branches, both of which supplied anterior wall of left ventricle. One important point to note is that all the three branches of left coronary artery were covered by left auricle and were exposed by retracting the left auricle.

## 3. Discussion

According to some authors, normal variant is an alternative pattern which is relatively infrequent compared to normal, but it occurs in more than 1% of otherwise normal individuals [2, 3]. The author discusses this case under four headings, namely, (1) anomalies of ostia, (2) courses, (3) termination of arteries, and (4) presence of myocardial bridge.

### 3.1. Anomalies of Ostia

The importance of ostium anomalies relates to the surgical difficulties encountered in cannulating these vessels during open aortic surgery or in performing coronary arteriography. The *number, location, level, and size of the ostium* are very important in successful performance of coronary angiogram [1]. 

#### 3.1.1. Anomalies in Number of Ostia

In multiple ostia, typically either the RCA or the conus branch arises separately, or the left anterior descending (LAD) and left circumflex (LCx) arteries are originated from different locations in the absence of LCA. Three or more ostia when these are located around sinus of valsalva are considered normal variants. Anomalous origin of 4 coronary ostia from the right sinus of Valsalva in a patient with hypertrophic cardiomyopathy was reported by Beach et al. [4]. The extra ostium due to conus branch arising directly from aorta is observed in 50% of cases. An aberrant conus artery arising separately from the RCA is particularly at risk for injury from ventriculostomy or other maneuvers performed during heart surgery [5]. Normally there is only one ostium in the left posterior aortic sinus for LCA. But multiple ostia were reported in the left posterior aortic sinus [2, 3, 6]. Separate ostia of the LCA and LCx artery occur in a small percentage, namely, (0.41%) by Danias et al. [7]and 0.5% to 8% of population [6]. 

In the present case, one ostium for LCA and two anomalously located (3 cm above the root of ascending aorta) additional ostia, for RCA and ACA are observed. Although multiple ostia represent a technical difficulty for the angiographer, they may also allow alternate collateral sources in patients with proximal coronary artery disease [8]. Multiple ostia may cause stasis of blood predisposing to thrombosis due to variation in hydrodynamic continuity.

#### 3.1.2. Anomalous Locations of Ostia in relation to the Normal Coronary Sinus

Variants of location of coronary ostium are described by many authors such as coronary artery that may arise from ostium situated at higher level at least one cm above the sinotubular junction instead of being at the aortic sinus [3, 9]. But in the majority of the cases, the positions of the ostia are below the sinotubular ridge [10]. Ostia above the sinotubular ridge [11, 12] have also been reported. 

 Under the present study the LCA originated from normally located aortic sinus. But RCA and ACA arose from separate ostia at the same level, situated 3.0 cm above root of aorta in anterior part far away from sinus valsalva. *The position, level, and size of the ostium, related to RCA and ACA in the present study, are different from one for RCA in normal configuration and studied by other authors*. Although these high level ostia are well tolerated and asymptomatic yet these may cause difficulty in cannulations during coronary angiography and cardiac bypass surgery [13, 14]. Difficulty in manipulating the catheter tips will be considerably higher in patients with the ostium above the level of STJ [1]. In the present study the ostia of RCA and ACA are very closely situated so they may further complicate the process of cannulation, and, chance of thrombosis increases due to alterations in hydrodynamic continuity modifying flow rate of blood. 

### 3.2. Anomalies of Course

Aberrant course taken by RCA and ACA observed here has not been described in the literature as far as known to the author. This aberrant course taken by these arteries has already been described in the case report section of this article. But anomalous conus artery (third coronary artery) originates from abnormally located ostium in aorta and it ends supplying the conus. But in this piece of work, the ACA having large extent not only supplies the conus, but also continues up to *left margin of left ventricle crossing aorta, infundibulum, thus supplying infundibulum and left ventricle* which are normally supplied by LCA. So *these configuration, extent, and area supplied by ACA are completely different. *If there is any disease because of atherosclerosis and spasm in the ACA, the structures supplied by it may suffer from ischemia. Diagnosis of ischemia in these structures may mislead the physician for defect in conus or LCA rather than ACA. Thus there may be misinterpretation in the diagnosis and management of heart diseases related to this variant course of ACA. 

 Trifurcation and quadrifurcation of LCA have been documented in the literature [15–18]. In the present case, LCA originates normally and anomalously trifurcates resulting in a prominent variant of courses of three branches of LCA through myocardium beneath left auricle. The anterior interventricular artery enters the groove and ends up near the inferior border. Since the anterior interventricular artery ends at the inferior border, therefore, the inferior surface which is supplied by this artery will be affected. Ramus intermedius after travelling for 2 cm entered into left ventricle. The marginal artery routing between the left auricle and left ventricle ended by making an arterial arcade with ACA. New branches of marginal artery may be additional source of supply to the left ventricle. Since the circumflex artery is absent, the area supplied by it may be affected. These variant coursings which may complicate the management and treatment of the disease may mislead the clinician. 

### 3.3. Presence of a New Variant of Myocardial Bridge

*Myocardial Bridging*. Normally the coronary artery is subepicardial. But when a portion of coronary artery is embedded in the myocardium, the myocardial tissues covering the artery form myocardial bridge. The artery which is covered by myocardium is known as “tunneled segment.” Myocardial bridge *is most commonly localized in the middle segment of the LAD artery *[19, 20]. 

In the present case *myocardial bridge* has been observed over *the main trunk of left coronary artery and its three branches.* The portion of anterior interventricular artery and ramus intermedius which are seen covered by myocardial tissues till these run below left auricle are exposed further. Marginal artery was entirely covered by myocardial bridge except a small portion which forms arterial arcade with the ACA. Myocardial bridging is described as protective by some authors while others link it with myocardial ischaemia, tachycardia-induced ischaemia, conduction disturbances, and myocardial infarction [3, 9, 14, 21]. In some cases, however, myocardial bridging is responsible for angina pectoris, myocardial infarction, life-threatening arrhythmias, or even death [19]. The standard of reference for diagnosing myocardial bridges is coronary angiography, at which a typical “milking” effect and a “step down-step up” phenomenon induced by systolic compression of the tunneled segment may be seen [22]. In contrast, multidetector row CT clearly shows the intramyocardial location of the involved coronary arterial segment [20]. The ECG-gated reconstruction window used in standard multidetector row CT of the coronary artery is usually positioned within the diastolic phase for maximal vasodilatation and minimal motion artefacts [23]. However, when there is suspicion for myocardial bridging, it is recommended that ECG-gated reconstruction be performed during the systolic phase as well as the diastolic phase. Comparison of the images obtained during the two phases will allow assessment of luminal narrowing during the systolic phase.

### 3.4. Arterial Arcade

ACA after travelling obliquely ends in making connection with marginal artery near the left border of the heart forming arterial arcade. There was also arterial arcade between the ACA and diagonal artery. Such arterial arcade has been described near the crux of the heart [8, 24, 25]. But the arterial arcade formed in the present case is not reported in the literature. Thus, in the present case a combination of variations is observed in the same specimen which is a *new finding.* Therefore if a cardiologist comes across one abnormality, he should also look for other abnormalities to fully understand the causatives of the problem. This will facilitate the proper diagnosis and treatment of cardiac diseases. The knowledge of the variations observed in the present study may be of paramount importance to cardiologists, radiologists, and anatomists.

## References

[1] Dombe DD, Anitha T, Giri PA, Dombe SD, Ambiye MV (2012). Clinically relevant morphometric analysis of left coronary artery. *International Journal of Biological and Medical Research*.

[2] Angelini P (1989). Normal and anomalous coronary arteries: definitions and classification. *American Heart Journal*.

[3] Angelini P (2002). Coronary artery anomalies—current clinical issues: definitions, classification, incidence, clinical relevance, and treatment guidelines. *Texas Heart Institute Journal*.

[4] Beach L, Burke A, Chute D, Virmani R (2001). Anomalous origin of 4 coronary ostia from the right sinus of valsalva in a patient with hypertrophic cardiomyopathy. *Archives of Pathology and Laboratory Medicine*.

[5] van Geuns RJ, Cademartiri F, Schoepf UJ (2005). Anatomy of the coronary arteries and vein in CT imaging. *CT of the Heart*.

[6] Patel S (2008). Normal and anomalous anatomy of the coronary arteries. *Seminars in Roentgenology*.

[7] Danias PG, Stuber M, McConnell MV, Manning WJ (2001). The diagnosis of congenital coronary anomalies with magnetic resonance imaging. *Coronary Artery Disease*.

[8] Greenberg MA, Fish BG, Spindola-Franco H (1989). Congenital anomalies of the coronary arteries: classification and significance. *Radiologic Clinics of North America*.

[9] Ko S-M, Choi J-S, Nam C-W, Hur S-H (2008). Incidence and clinical significance of myocardial bridging with ECG-gated 16-row MDCT coronary angiography. *International Journal of Cardiovascular Imaging*.

[10] Turner K, Navaratnam V (1996). The positions of coronary arterial ostia. *Clinical Anatomy*.

[11] Vlodaver Z, Neufeld HN, Edwards JE (1975). *Coronary Arterial Variations in the Normal Heart and in Congenital Heart Disease*.

[12] Pejković B, Krajnc I, Anderhuber F (2008). Anatomical variations of coronary ostia, aortocoronary angles and angles of division of the left coronary artery of the human heart. *Journal of International Medical Research*.

[13] Dodd JD, Ferencik M, Liberthson RR (2007). Congenital anomalies of coronary artery origin in adults: 64-MDCT appearance. *American Journal of Roentgenology*.

[14] Montaudon M, Latrabe V, Iriart X, Caix P, Laurent F (2007). Congenital coronary arteries anomalies: review of the literature and multidetector computed tomography (MDCT)-appearance. *Surgical and Radiologic Anatomy*.

[15] Baptista CAC, DiDio LJA, Prates JC (1991). Types of division of the left coronary artery and the ramus diagonalis of the human heart. *Japanese Heart Journal*.

[16] Cavalcanti JS (1995). Anatomic variations of the coronary arteries. *Arquivos Brasileiros de Cardiologia*.

[17] Reig J, Petit M (2004). Main trunk of the left coronary artery: anatomic study of the parameters of clinical interest. *Clinical Anatomy*.

[18] Hirak D (2005). Termination of left coronary in the population of Assam. *National Journal of Basic Medical Sciences*.

[19] Tio RA, van Gelder IC, Boonstra PW, Crijns HJGM (1997). Myocardial bridging in a survivor of sudden cardiac near-death: role of intracoronary Doppler flow measurements and angiography during dobutamine stress in the clinical evaluation. *Heart*.

[20] Amoroso G, Battolla L, Gemignani C (2004). Myocardial bridging on left anterior descending coronary artery evaluated by multidetector computed tomography. *International Journal of Cardiology*.

[21] Leschka S, Koepfli P, Husmann L (2008). Myocardial bridging: depiction rate and morphology at CT coronary angiography—comparison with conventional coronary angiography. *Radiology*.

[22] Mohlenkamp S, Hort W, Ge J, Erbel R (2002). Update on myocardial bridging. *Circulation*.

[23] KoppAF, Schroeder S, Kuettner A (2001). Coronary arteries: retrospectively ECG-gated multi-detector row CT angiography with selective optimization of the image reconstruction window. *Radiology*.

[24] Kim SY, Seo JB, Do KH (2006). Coronary artery anomalies: classification and ECG-gated multi-detector row CT findings with angiographic correlation. *Radiographics*.

[25] Kruskal JB, Hartnell GG (1995). Nonatherosclerotic coronary artery disease: more than just stenosis. *Radiographics*.

